# From the gut to the brain: The involvement of the gut microbiota in the development and progression of glioblastoma

**DOI:** 10.1093/noajnl/vdaf267

**Published:** 2025-12-22

**Authors:** Daniela Toumazi, Christiana Charalambous, Constantina Constantinou, Nicoletta Nicolaou

**Affiliations:** Department of Basic and Clinical Sciences, University of Nicosia Medical School, Nicosia, Cyprus; Department of Basic and Clinical Sciences, University of Nicosia Medical School, Nicosia, Cyprus; Department of Basic and Clinical Sciences, University of Nicosia Medical School, Nicosia, Cyprus; Department of Basic and Clinical Sciences, University of Nicosia Medical School, Nicosia, Cyprus

**Keywords:** antibiotics, glioblastoma, gut microbiota, gut-brain axis, probiotics

## Abstract

Glioblastoma (GB) is the most malignant tumor in the adult central nervous system (CNS), presenting substantial treatment challenges due to its infiltrative nature, heterogeneity and immunosuppressive environment it creates. Current therapeutic efforts are focused on enhancing our understanding of GB and developing effective therapies. An emerging area of interest is the bidirectional gut–brain axis, which mediates communication between gut microbiota and CNS. The gut–brain axis allows the microbiota to modulate the immune system and inflammatory pathways through microbial metabolites, such as short-chain fatty acids (SCFAs) and tryptophan derivatives, promoting or suppressing GB progression. Understanding these interactions can lead to microbiota-targeted therapies for GB patients. Novel therapies, such as fecal microbiota transplantation to enhance immunotherapy response and using bacterial toxins to cross the blood–brain barrier, show promise in improving treatment-resistant GB treatment. Additionally, the role of probiotics and antibiotics on GB prognosis is being investigated. While more research is needed to understand the gut microbiota’s role in GB, recent findings suggest promising directions for future therapies. This review examines the interplay between key immune system components and the microbiota in GB development and explores how this understanding could facilitate the development of novel therapeutic interventions.

Key Points

- Glioblastoma (GB) is challenging to treat due to its complex pathogenesis and immunosuppressive environment. The gut–brain axis and microbiota affect GB development, offering potential for new therapies including fecal transplant and probiotics.

Glioblastoma (GB) is the most common type of gliomas in adults^1^ and one of the most malignant types of CNS tumors, accounting for 3%-4% of all cancer-related deaths, with a 5-year survival rate of 4%-5% and a median survival of 12.6 months.^2^ The GB global incidence is 10 per 100,000 population and the incidence normally increases with age.^1^^,^^3^

The pathogenesis of brain tumors is complex and not fully understood, contributing to poor prognosis and less effective treatments compared to other cancers. While the exact mechanisms of GB pathogenesis remain elusive, genomic advancements have shed light on critical molecular alterations associated with GB. Key genetic changes include mutations and amplification of the *EFGR* gene, deletion of the *p16* gene, and loss of heterozygosity on chromosome 10q. Additionally, mutations in the *PTEN* and *TERT* promoters are frequently observed.^3^

Proinflammatory cytokines, especially interleukin-6 (IL-6) and interleukin-8 (IL-8), play a significant role in the initiation and progression of brain tumors.^4^ IL-6 activates pathways involving the transcription factors nuclear factor kappa B (*NF-κB*) and signal transducer and activator of transcription 3 (*STAT-3*).^4^ IL-8 is one of 2 pathways that control angiogenesis in human gliomas in a paracrine manner (the second pathway is mediated by vascular endothelial growth factor (VEGF) and/or fibroblast growth factor).^5^ This activation enhances tumor aggressiveness by promoting cellular processes such as proliferation, differentiation, and survival. Studies have indicated that inhibiting IL-6, STAT-3, and NF-κB can effectively reduce glioma growth.^6^^,^^7^

Standard management for GB is surgical resection followed by chemotherapy, specifically temozolomide (TMZ).^8^ However, even with aggressive treatment, 90% of patients experience tumor recurrence.^8^^,^^9^ Several factors contribute to the difficulty in treating GBs. The high infiltrative nature of GB makes complete surgical resection almost impossible, while its significant intertumor and intratumor heterogeneity complicates targeted therapy. Additionally, GB’s immunosuppressive microenvironment, often referred to as a “cold tumor” due to its lack of tumor antigens, defects in antigen presentation, and high concentration of immunosuppressive cells hinders a strong T cell response.^10^ This makes management with immune checkpoint inhibitors (ICI), such as anti-CTLA-4 and anti-PD1/PD-L1, particularly challenging.^11^^,^^12^ Given GB’s poor prognosis and aggressive nature, developing more effective and innovative therapies is of outmost importance.

The gut–brain axis is a complex, bidirectional communication network that links the gastrointestinal tract and the CNS. It involves multiple overlapping systems, including the autonomic (ANS) and enteric (ENS) nervous systems, the neuroendocrine system, and the immune system.^13^ A central player in this axis is the gut microbiota, a vast and diverse ecosystem of trillions of microorganisms (including bacteria, viruses, and fungi), which modulates these pathways through a variety of biochemical and immune mechanisms.^14^

The microbiota influences the gut–brain axis both directly and indirectly through the production of microbial and host-derived metabolites, as well as neurotransmitters ­(Figure 1). For example, microbial metabolites, such as short-chain fatty acids (SCFAs), can cross the gut epithelial barrier and enter the bloodstream, where they impact immune regulation and brain function. Similarly, neurotransmitters, such as serotonin and gamma-aminobutyric acid (GABA), synthesized by gut bacteria, are known to influence mood, behavior, and cognition. The immune system also serves as another crucial link, as gut microbial signals shape immune cell function, contributing to either neuroinflammation or neuroprotection.^15^

**Figure 1.**
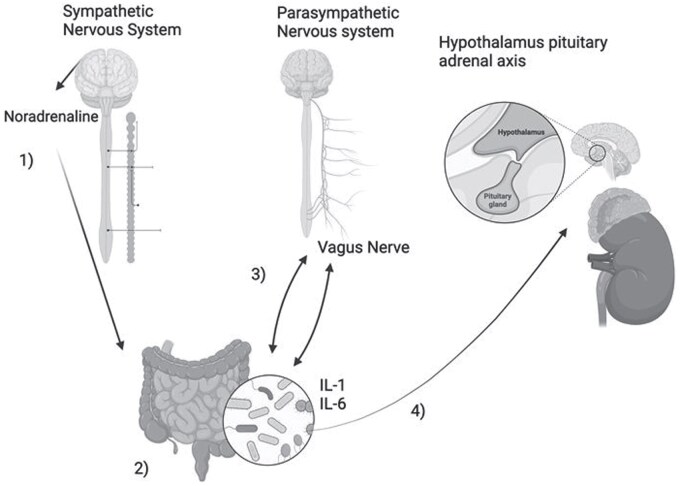
The gut–brain axis. (1) Noradrenaline, a known neurotransmitter from the sympathetic pathway, promotes bacterial growth in the gut and affects barrier function. (2) The gut microbiota is able to affect gut motility indirectly by activating the toll-like 4 receptors found in enteric glial cells. (3) The vagus nerve acts as a bidirectional communication track between the gut microbiota and the brain allowing metabolites of the microbiota to affect the CNS directly. (4) Enteropathic *Escherichia coli* bacteria secrete IL-2 and IL-6, which act on the adrenal glands and are considered to be the most potent activators of the HPA axis ,resulting in subsequent secretion of cortisol. Created with BioRender (access link: *Final share link will be provided upon publication, as per BioRender academic license requirements*).

Recent research has highlighted the impact of the gut–brain axis on various brain disorders, including neurodegenerative disorders and psychiatric conditions,^16^^,^^17^ and brain tumors.^18–20^ In cancer, the diversity and abundance of specific microbial species can affect cancer susceptibility and response to treatment by modulating systemic inflammation, immune responses, and alteration of metabolic pathways. Disruptions in gut microbiota composition, or dysbiosis, have been linked to an increased risk of certain types of cancers including colorectal, gastric and breast cancers.^21^^,^^22^ For instance, certain bacteria can metabolize dietary components into carcinogens or anti-carcinogens,^23^ influencing tumor development. In colorectal cancer, the microbiome is known to influence carcinogenesis^24^ and patient response to chemotherapy.^25^ Similarly, in melanoma, the gut microbiota has been shown to modulate the efficacy of immunotherapies, such as checkpoint inhibitors, by affecting systemic immune responses.^26^ These findings suggest that the gut microbiome can play a role in shaping the tumor microenvironment and influencing treatment outcomes across different cancers.

Emerging evidence links gut microbiota to immune modulation in GB, contributing to its immunosuppressive environment.^27^^,^^28^ Exploring the gut–brain axis in cancer may offer new therapies. Strategies to adjust the gut microbiome, such as diet, probiotics, prebiotics, or fecal microbiota transplantation, aim to correct dysbiosis, modulate immunity, and reduce tumor-promoting inflammation.

The aim of the current review is to update the current understanding of the association between key immune system components and the microbiota in the development of GB, and to explore how this knowledge can inform the development of effective treatments for managing the disease.

## Methods

A literature search was performed in PubMed and ScienceDirect databases using the keywords “Glioblastoma multiforme,” “gut microbiota,” “gut-brain axis,” “antibiotics,” “pro­biotics,” “immunotherapy,” “microbiota metabolites,” “blood brain barrier,” “glioma-associated macrophages,” “Temozolomide,” “neurotransmitters,” “immune check-point inhibitors,” and “fecal microbiota transplantation.” Inclusion criteria: peer-reviewed original research studies (*in vitro* and *in vivo* in animals and humans), systematic reviews and meta-analyses, published in the English language during the period 2011-2025. Exclusion criteria: scoping reviews or published in languages other than the English language. Reference lists from articles were also manually reviewed to include additional relevant articles. Titles and abstracts were reviewed to determine study eligibility. The selected articles were stratified to answer the main research question of the associations between the gut microbiota and the development and progression of GB.

## The Role of the Immune System in GB Carcinogenesis

The immune system plays a crucial role in GB tumorigenesis, particularly in shaping its highly immunosuppressive tumor microenvironment. One of the major challenges in GB management is its ability to evade immune surveillance through various mechanisms, including the recruitment of immunosuppressive cells, alterations in cytokine signaling, and disruption of the blood–brain barrier (BBB). Understanding these immune functions within the tumor microenvironment is critical for developing novel immunotherapeutic strategies.^7^  Table 1 shows a summary of the contribution of various immune cells to the development of GB.

**Table 1.** The role of specific immune cells in the development of GBM

Type of immune cells	Description of immune cells	Role in GBM development	Reference
Glioma- associated macrophages (GAMS)	GAMs are either myocytes derived from bone marrow or microglia that reside in the brain	↑ TGF-β, IL-6 and IL-1β ↑permeability of BBB ↑ tumour proliferation and growth	^29^
T helper (TH) cells	T helper cells are crucial in maintaining immunological balance in CNS Th1- produce proinflammatory cytokines Th2- produce antiinflammatory cytokines	Th2 ↑ IL-6 and TGF-β⟹ favors immunosuppressive environment IL-6 STAT signaling pathway ↑ cell cycle progression and angiogenesis and ↓ apoptosis ↑ IL-6 associated with poor prognosis in GBM patients. ↓ IL-6 STAT signaling pathway associated with decrease in glioma growth	^7^ ^30^
Regulatory T cells (Tregs)	Tregs are a subset of CD4+ T lymphocytes and contribute to immunosuppressive environment of GBM	Produce IL-10 and TGF-β⟹ IL-10 ↓ T cell proliferation ↓ Tregs associated with ↑survival in GBM	^29^ ^29^ ^,^ ^30^

Abbreviations: BBB: *blood–brain barrier*; GBM: *glioblastoma*; VEGF: *vascular endothelial growth factor*; Th: *T helper cell*.

Emerging evidence suggests that the gut microbiota significantly influences immune system function in GB by shaping myeloid and lymphoid cell responses. Microbial metabolites such as SCFAs and tryptophan derivatives can promote either proinflammatory or immunosuppressive immune states, potentially affecting GB progression and therapy response.^10^^,^^31^ SCFAs, particularly butyrate, have been shown to suppress proinflammatory pathways via inhibition of NF-κB, which plays a key role in glioma-related immune suppression. Conversely, microbial kynurenine metabolism leads to the activation of the aryl hydrocarbon receptor (AHR), further skewing the immune environment toward immunosuppression by increasing regulatory T cell (Treg) activity.^32^ These findings suggest that gut microbiota alterations could directly influence the tumor immune microenvironment, contributing to immune evasion and therapy resistance in GB.

The BBB is a highly specialized, selective barrier formed by endothelial cells with tight junctions, astrocyte end-feet, and pericytes, which collectively regulate the passage of substances between the bloodstream and the central nervous system (CNS).^8^^,^^10^ Traditionally, the brain has been described as “immune privileged” due to the restricted entry of peripheral immune cells.^29^ However, recent studies suggest that the brain should instead be considered “immune distinct,” accounting for its specialized resident immune components, including microglial cells, T helper cells, regulatory T cells and natural killer cells (NK cells).^10^

In GB, the integrity of the BBB is significantly compromised. This disruption, which is characterized by poorly formed blood vessels, upregulation of transporter proteins, and downregulation of tight junction proteins, leads to increased permeability.^33^^,^^34^ This, in turn, allows for the infiltration of peripheral immune cells, including T cells, tumor-associated macrophages, and myeloid-derived suppressor cells,^8^ contributing to the immunosuppressive microenvironment characteristics of GB.^10^^,^^35^ Additionally, the increased BBB permeability enables the entry of various growth factors and cytokines that further promote tumor invasion, angiogenesis, and proliferation.^34^^,^^36^ Thus, the enhanced permeability of the BBB in GB exacerbates tumor progression and poses significant challenges for effective drug delivery, as therapeutic agents must navigate the altered barrier to reach the tumor site.^29^

### Microglia and Glioma-Associated Macrophages

Microglia are CNS-resident myeloid cells that support immune surveillance and tissue homeostasis.^29^ They regulate immunity through cytokine production, phagocytosis, and antigen presentation.^37^ Microglia can respond to external stimuli by shifting between M1 (proinflammatory) and M2 (antiinflammatory or immunosuppressive) phenotypes.^7^ M1 microglia produce TNF-α, IL-6, and IL-1β that enhance antigen presentation, cytotoxic T-cell activity, and tumor cell killing,^7^^,^^35^ whereas M2 microglia secrete IL-10, TGF-β, EGF, and VEGF that promote tissue repair, angiogenesis, and tumor progression.^10^

Under physiological conditions, a dynamic balance between M1 and M2 microglia is maintained, allowing for both immune defense and tissue repair as needed.^38^ However, in GB, the tumor microenvironment skews this balance toward the M2-dominant phenotype.^10^ Recent evidence suggests that gut microbiota-derived metabolites, particularly SCFAs including butyrate, directly influence microglial polarization by promoting an M2-like antiinflammatory phenotype, which may exacerbate the immunosuppressive microenvironment in GB.^32^ Butyrate suppresses proinflammatory responses by inhibiting histone deacetylases (HDACs) and reducing NF-κB activation, a key regulator of microglial inflammatory pathways.^31^ Clinically, a high M2: M1 ratio in GB correlates with poor prognosis,^39^ while increased levels of M2 are inversely correlated with patient survival time.^7^^,^^40^

Glioma-associated macrophages (GAM) comprise both resident microglia and infiltrating macrophages derived from bone marrow.^35^ Similarly to microglia, these macrophages are highly plastic and respond to the GB microenvironment by predominantly adopting an M2-like phenotype; by secreting TGF-β, IL-6, and IL-1β they promote invasion, increase BBB permeability, and accelerate tumor growth.^35^^,^^41^ Clarifying and therapeutically redirecting M1/M2 polarization, including microbiome-influenced pathways, may shift these cells toward antitumor activity.

### T-Helper Cells

In GB, the CNS immune balance is disrupted, creating an immunosuppressive environment that supports tumor progression.^42^ T-helper cells (Th1 and Th2) are crucial for maintaining CNS immune homeostasis.^43^ More specifically, Th1 cells produce proinflammatory cytokines, such as TNF-α and IL-1β, contributing to antitumor responses through macrophage and cytotoxic T cell activation. In contrast, Th2 cells produce antiinflammatory cytokines (eg IL-4, IL-5, and IL-10), typically associated with immune suppression and tumor tolerance.^30^

There is growing evidence that the gut microbiota directly influences Th1/Th2 polarization through the activity of specific microbial metabolites. SCFAs, such as butyrate and propionate, have been shown to modulate CD4^+^ T cell differentiation, promoting IL-10 production by Th1 cells and supporting antiinflammatory responses via GPR43 signaling.^44^ Other microbial-derived molecules, such as γ-polyglutamic acid (γ-PGA), can bias dendritic cell activation in a way that favors Th1 over Th2 responses.^45^ Altered microbiota composition has also been linked to dysregulated CD4+ T-cell profiles in CNS disorders in mouse models,^46^ suggesting that microbiota-derived signals may influence neuroimmune environments relevant to GB. Additionally, broader analyses have shown that dietary and microbiota-derived metabolites can shape the balance of T-helper subsets, with implications for systemic and CNS immune responses.^47^^,^^48^ While these findings are largely derived from intestinal and systemic immune models, they provide a compelling framework for understanding how gut-derived signals may skew T-helper cell phenotypes in the GB microenvironment.

In GB, this shift may contribute to tumor-promoting immunosuppression. Recent studies suggest that gut-derived SFCAs and tryptophan metabolites bias CD4+ T cells away from Th1 and toward Th2 phenotypes, driving M1 microglia/macrophage polarization toward the tumor-promoting M2 phenotype.^49^^,^^50^ Th2-secreted cytokines, such as IL-6, enhance tumor cell proliferation, angiogenesis, and inhibit apoptosis via STAT3 signaling, a pathway associated with GB aggressiveness and poor clinical outcomes.^51^ In contrast, Th1-secreted cytokines are markedly reduced in GB, creating an imbalance that favors an immunosuppressive environment,^7^ supporting tumor development and immune evasion.

Thus, gut microbiota-mediated modulation of Th1/Th2 balance may be a key mechanism linking gut dysbiosis to GB progression, highlighting a potential target for therapeutic intervention.

### Regulatory T Cells

Regulatory T cells (Tregs) are a specialized subset of CD4+ T lymphocytes, which inhibit T cell proliferation and enhance immunosuppression through the secretion of antiinflammatory cytokines, such as TGF-β and IL-10.^7^^,^^10^ Gut microbiota-derived metabolites promote and stabilize these Treg programmes. For example, microbial SCFAs enhance expression of FOXP3 via HDAC inhibition and promote Treg differentiation.^45^^,^^52^ Similarly, gut-derived tryptophan metabolites (such as indoles) activate the AHR pathway and contribute to Treg stability and function.^53^ In germ free (GF) or dysbiotic models, Treg populations are significantly reduced, underscoring the microbiota dependency of this axis.^54^ In the context of GB, such metabolite-driven expansion or activation of Tregs may contribute to the immunosuppressive tumor microenvironment, support M2-skewed microglia and macrophages, dampen antitumor immunity, and thereby facilitate invasion, angiogenesis, and tumor growth.^55^^,^^56^

In GB, the presence of Tregs is associated with poor prognosis and higher tumor grades.^10^ Studies have shown that depleting Tregs in GB-implanted mice leads to tumor rejection and prolonged survival, highlighting the potential therapeutic benefit of targeting Tregs in GB treatment.^10^ These findings suggest that further studies are needed to explore strategies for selectively reducing Treg levels or inhibiting their function within the tumor microenvironment, potentially enhancing immunotherapy efficacy and inhibiting GB progression.

## The Role of Microbiota and Their Metabolites in the Development of Glioblastoma

Gut microbiota contributes to GB progression by producing a variety of immune-modulating and neuroactive compounds. These include SCFA, tryptophan derivatives, bacterial toxins, and neurotransmitter-like molecules that interact with host immune cells and neural elements within the tumor microenvironment. Table 2 provides an overview of selected microbiota-derived metabolites and bacterial factors, their microbial producers, and their proposed effects on the GB microenvironment, including the primary host cell types they are known or hypothesized to influence.

**Table 2.** Microbiota-derived compounds implicated in glioblastoma progression, with associated microbial sources, functional effects, and primary effector cell targets

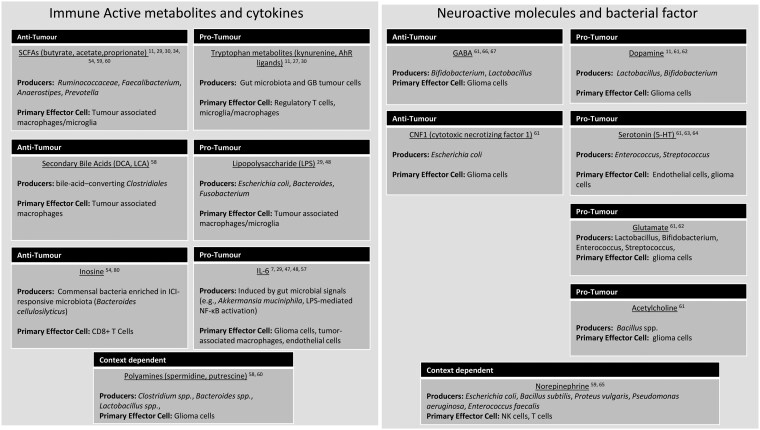

### The Effect of the Microbiota on Glioma-Neural Interactions

Emerging evidence suggests that the gut microbiota plays a direct role in modulating glioma-neural interactions, influencing tumor progression through multiple mechanisms. In a recent study, Li et al.^0^ have identified specific bacterial genera, such as *Fusobacterium*, within glioma tissues, where their presence has been linked to the regulation of synapse-related gene expression. This suggests that intratumoral microbiota may influence neuronal communication and glioma progression by modulating tumor-neuron interactions at the molecular level.^60^

Furthermore, the gut microbiota has been shown to regulate microglia maturation and function, which is crucial for synaptic transmission and neuronal circuit formation. In GF mice, microglia exhibit an immature phenotype and impaired responses to environmental challenges, indicating that gut microbiota-derived signals are necessary for proper microglial function.^61^ Given the increasing recognition that glioma cells form synaptic-like interactions with neurons, such as glutamatergic tumor–neuron synapses,^62^^,^^63^ and that the gut–microbiota–brain axis can modulate CNS immune function, glial activity, and neurotransmitter signaling,^18^^,^^64–67^ it is plausible that gut-derived microbial metabolites and immune modulation may influence glioma growth by shaping the tumor microenvironment through microglial regulation and neuronal signaling circuits. Collectively, this emerging research highlights a potential gut–brain–tumor axis, where gut microbiota modulates glioma–neural interactions.

### Gut Microbiota and Dysbiosis

The gut microbiota plays a crucial role in maintaining host health and modulating immune responses. Dysbiosis, an imbalance in the gut microbiota composition, has been implicated in the development and progression of various cancers, including colorectal, breast, and gastric cancers.^22^ Recent studies indicate that gut microbiome dysbiosis contributes to the immunosuppressive environment in GB and its progression.^10^ Emerging evidence supports differences between the gut microbiomes of GB patients and healthy controls, linking gut dysbiosis with the disease.

In a study by Jiang et al.^8^ 16S rRNA gene sequencing of fecal samples from GB patients and controls revealed reduced microbial diversity and significant alterations in the gut microbiota of GB patients. Notably, there was an increase in pathogenic bacteria from the *Fusobacteria* and *Bacteroidetes* phyla, alongside a decrease in beneficial ­bacteria from the *Firmicutes*, *Actinobacteria*, and *Verrucomicrobia* phyla. The study identified 6 bacterial biomarkers—*Fusobacterium*, *Escherichia/Shigella*, *Ruminococcus gnavus* group, *Lachnospira*, *Akkermansia*, and *Parasutterella—*capable of distinguishing GB patients from healthy individuals. These findings suggest the potential of microbiota-based diagnostics for GB.

### Metabolites

Beyond shifts in the gut microbiome composition, gut microbiota-derived metabolites directly influence the tumor microenvironment by modulating immune responses and inflammatory pathways. Although the exact mechanisms remain unclear, several mechanisms have been proposed to explain how gut microbiota may influence GB development.

First, gut microbiota modulates the host immune system through the production of metabolites, such as SCFAs (butyrate, acetate, and propionate), which suppress proinflammatory cytokines and promote Treg expansion.^32^ Dysbiosis, characterized by reduced SCFA-producing bacteria, increases proinflammatory cytokine levels, such as IL-6 and TNF-α, enhancing angiogenesis and suppressing antitumor responses.^31^ Thus, dysbiosis can lead to an imbalance in these immune cell populations, promoting an immunosuppressive environment favoring GB progression.^69^

Second, gut bacteria can directly produce and modulate the levels of various cytokines, such as IL-6 and TNF-α, which play critical roles in inflammation and tumorigenesis.^4^ Elevated levels of proinflammatory cytokines can enhance tumor growth by promoting angiogenesis and suppressing antitumor immune responses.^70^

Third, the gut–brain axis allows gut microbiota to influence brain function and pathology via neural, endocrine, and immune pathways, promoting GB progression.^66^ Dysbiosis disrupts this communication, leading to alterations in brain function, and can also lead to increased permeability of the BBB, allowing circulating immune cells and microbial metabolites to infiltrate the CNS and interact with the tumor microenvironment.^71^ Microbial metabolites produced by gut microbiota, such as tryptophan derivatives and secondary bile acids, can cross the BBB and affect brain function and pathology.^72^

Additionally, gut microbiota influences the metabolism of tryptophan, leading to the accumulation of kynurenine, which interacts with the AHR pathway, driving immune suppression and GAM polarization toward an M2-like phenotype.^27^ Several studies have demonstrated these immunomodulating effects of gut bacteria and their metabolites on disease progression.^6^^,^^57^^,^^58^  Table 3 summarizes relevant studies that collectively highlight the complex interactions between gut microbiota and GB, emphasizing the potential for microbiota-based therapeutic strategies to influence GB progression and treatment outcomes.

**Table 3.** Summary of studies investigating the role of microbiota in GBM in animal and human studies

Animal studies				
Study	Aim	Type of study and methodology	Key findings	Main conclusion
Patrizz et al.	To investigate the effects of glioma development and TMZ on the fecal microbiome in mice.	**Mouse glioma model** **Methodology:** C57BL/6 mice implanted with GL261/sham and administered with TMZ or saline. Fecal samples collected and analyzed using 16S rRNA sequencing.	↑ dysbiosis in gut microbiome following glioma development. Without TMZ: ↑*Verrucomicrobia* phylum and *Akkermansia* genus ↓*Firmicutes* phylum and *Firmicutes: Bacteroides* ↓With TMZ- no changes in microbiota TMZ prevented glioma induced dysbiosis.	Glioma development results in gut dysbiosis in mouse model. Findings were not observed following administration.
D’Alessandro et al.^3^	To investigate the effects of antibiotics on intestinal microbiota following glioma growth in mice.	**Mouse glioma model** **Methodology:** C57BL/6N mice implanted with GL261 and treated with antibiotics, vancomycin and gentamicin and sucralose. Control group was given sucralose alone. Cell isolation and flow cytometric analyses and tumour volume evaluation.	Chronic treatment of antibiotics ⟹ ↑ tumour growth in mice implanted with GL261 and antibiotics Modified gut microbiota in mice treated with antibiotics °↑*Burkholderiales* °↓*Prevotellaceae, Rikenellacaea, helcobacteraceae* ↓ cytotoxic NK cell subsets in mice treated with antibiotics. ↓ frequency of CD27+/CD11b+ cell subset in mice treated with antibiotics.	Chronic antibiotic treatment in mouse model alters the gut microbiota, reduces cytotoxic NK cell subsets, and impairs microglia functions which affect Glioma growth in mice.
Dees et al.^64^	To investigate the role of human microbial communities on the growth and response to immunotherapy in a preclinical mouse model of glioma.	**Mouse Glioma Model** **Methodology:** Mouse Glioma model (GL261) transplanted with human microbial communities from 5 healthy donors. All 5 humanized mouse lines (HuM1-5) were susceptible to GBM transplantation. Mice transplanted with healthy human donor fecal samples and were either given immunotherapy (anti PD1) or isotype control.	HuM 2 and 3 °Good response to anti-PD1 °↓ tumour growth and increased survival. °Both showed similar microbial communities HuM1,4,5 °Poor response to PD1 °No survival benefit HuM 2 and 3 °↑*Bacteroides cellulosilyticus,* HuM 2, 4 and 5 °↑*Blautia producta* *HuM 1* °↑*Bacteroides ovatus* *HuM 4 and 5* ↑*Bacteroides intestinalis, Bacteroides uniformis*	Human microbiota that was implanted in the GI tract of mice influenced the response of immunotherapy in the context of GBM. Some microbiomes, such as those rich in *Bacteroides cellulosilyticus,* showed a positive response to anti- PD1 therapy while other microbiomes were nonresponsive.
Dono et al.^2^	To examine fecal SCFAs and neurotransmitter alterations in mice	**Mouse glioma model** **Methodology:** C57BL/6 mice transplanted with GL261 and with either TMZ or saline. Fecal samples collected to measure metabolite levels and taxa abundance. Liquid chromatography-mass spectrometry and 16s rRNA-sequencing was performed.	↓ norepinephrine and 5-HIAA ↓SCFAs ↓ in other neurotransmitters TMZ ↓ fecal metabolite changes in mice ↑*Bacteroides, Verrucomicrobia, akkermansia*	In glioma bearing mice, levels of 5-HIAA and norepinephrine decreased. SCFAs levels decreased

Human studies				
Study	Aim	Type of study and methodology	Key findings	Main conclusion
Patrizz et al.	To investigate effects of glioma development and Temozolomide (TMZ) on the fecal microbiome in humans.	**Prospective cohort study** **Methodology:** Fecal samples collected from healthy controls and 53 newly diagnosed glioma patients before and after chemoradiation (75% had GBM- *IDH-WT,* 12% had GBM *IDH-mutant)* Fecal samples analysed using 16S rRNA sequencing.	Similar changes observed in microbiota in glioma patients. TMZ associated dysbiosis in glioma patients *Without TMZ* ↑*Bacteroidetes, Proteobacteria, Verrucomicrobia, akkermansiaceae akkermansia (seen in IDH-WT)* ↑*Firmicutes: bacteroides (both)* *Firmicutes and Actinobactera (seen in IDH-WT* *With TMZ* ↑*Akkermansia, Verrucomicrobia, Akkermansiaceae*	*Akkermansia Verrucomicrobia* increased after glioma growth in humans with IDH-WT gliomas but was not seen when TMZ was administered, showing the relationship between gliomas, gut brain axis, and TMZ.
Dono et al.^9^	To examine feacal SCFAs and neurotransmitter alterations in glioma patients.	**Prospective study** **Methodology:** Fecal samples were obtained from a cohort of glioma patients and healthy controls. liquid chromatography-mass spectrometry and 16s rRNA- sequencing performed.	↓norepinephrine and 5-HIAA without TMZ No significant changes to fecal metabolites with TMZ	In humans with glioma, 5-HIAA and norepinephrine decreased
Haedenkamp^84^	To investigate the relationship between the use of antibiotics and risk of developing glioma in a case control study	**Case control study** **Methodology:** 4423 glioma cases involved in a conditional logistic regression analysis. Exposures of interest were the use of any antimicrobial drugs: Antimicrobials Antivirals Antifungals Antiprotozoals Antihelmintics	No clear evidence to support increased risk of developing gliomas after antibiotic usage	Antibiotic use does not increase the risk of glioma development.

Abbreviations: 5-HIAA, *5-hydroxyindoleacetuc acid*; SCFAs, *short-chain fatty acids*; TMZ, *temezolomide*.

In an *in vivo* study by Patrizz et al.the effects of glioma development and TMZ on the fecal microbiome of mice and humans were explored.^6^ Mice implanted with GL261/sham (a murine glioma model) showed significant bacterial dysbiosis, evidenced by a decreased *Firmicutes: Bacteroides* ratio and an increase in *Akkermansia muciniphilia*. *A. muciniphila* has been implicated in proinflammatory pathways that may lead to the breakdown of the intestinal membrane and facilitate communication between peripheral immune cells and the microbiota.

*A. mucinophilia* also exhibits immunoregulatory effects by activating TLR-2, inducing the release of cytokines from peripheral blood mononuclear cells and activating NF-κβ.^73^ The activation of NF-κβ is thought to induce the STAT3 signaling pathway, promotes cell cycle progression, angiogenesis, and immune evasion and prevents apoptosis in gliomagenesis.^51^ Aberrant activation of NF-κβ in GB is linked to cancer cell invasion and resistance to radiotherapy.^6^ Similar results (ie a change in the *Firmicutes: Bacteroides* ratio and an increase in *A. muciniphilia*) were observed in glioma patients, most of whom were diagnosed with GB IDH-wildtype.^6^ Notably, the increased abundance of *A. mucinophilia*, was not observed following TMZ treatment both in mice and in glioma patients. While this study demonstrated a relationship between the gut–brain axis, glioma development and TMZ, further research is needed to understand the gut microbiome’s role in GB development.

Depending on the presence or absence of specific bacteria, dendritic cells can activate Th1 and Th17 cells to produce proinflammatory cytokines. *B. fragilis* plays a key role in differentiating Tregs that secrete IL-10, which increases glioma progression and aggressiveness by impairing anticancer Th1 immunity.^7^ Additionally, specific types of microbiota can enhance immune suppression, a critical factor in GB progression.^7^ Therefore, manipulating the composition of the gut microbiota to favor microbes that improve host immunity is crucial.^7^ Interestingly, GF mice exhibit altered microglial morphology and gene expression profiles, suggesting that gut microbiota plays a significant role in microglial maturation and function.^37^

Two recent Mendelian randomization studies explored the causal relationship between gut microbiota composition and GB risk.^32^^,^^74^ Wang et al.^4^ found that an increased abundance of the bacterial family *Ruminococcaceae* was linked to a reduced risk of GB, suggesting a protective effect, while the family *Peptostreptococcaceae* and the genus *Eubacterium* brachy group were associated with higher GB risk, suggesting that enhancing *Ruminococcaceae* levels could be clinically significant in reducing GB risk. In the study by Zeng et al.^32^  *Anaerostipes*, *Faecalibacterium*, *Prevotella7*, and *Ruminococcaceae* were identified as microbial taxa with protective effects against GB. Notably, *Prevotella7* showed a bidirectional causal relationship with GB, indicating its abundance may influence GB development and vice versa. This study also supported findings that an increased abundance of the *Eubacterium* brachy group is linked to higher GB risk.^32^

These studies highlight the complex role of gut microbiota in GB pathogenesis, emphasizing its potential protective effects and the need for further research. Understanding these mechanisms could lead to new preventive and therapeutic strategies. Gut microbiota dysbiosis not only changes microbial composition but also contributes to the immunosuppressive environment in GB, potentially affecting treatment resistance.

### Gut Microbiota-Mediated Modulation of GB

The complexity of gut microbiota-host interactions in GB extends beyond immunomodulation. Increasing evidence highlights the gut microbiota’s ability to modulate inflammatory pathways through its metabolic by-products. Dysbiosis-induced disruption of metabolites such as SCFAs, bile acids, tryptophan derivatives, and polyamines significantly influence glioma-associated inflammation.^74^

SCFAs (butyrate, acetate, and propionate) are well-characterized microbiota-derived antiinflammatory metabolites that modulate immune responses through HDAC inhibition and G-protein-coupled receptor activation. By reducing nuclear factor-κB (NF-κB) signaling, SCFAs suppress the secretion of proinflammatory cytokines (IL-6, TNF-α, IL-1β), thereby mitigating tumor-promoting inflammation in GB.^69^ SCFAs also regulate IL-10 and TGF-β levels and are involved in the polarization of microglia into the M2 phenotype.^35^ Additionally, SCFAs regulate T-cell differentiation, enhancing regulatory Treg expansion while reducing pro-tumourigenic Th17 responses,^35^^,^^59^ which are implicated in GB-associated immunosuppression.^32^ The dysbiosis-related decline in SCFA-producing bacteria tips the immune balance toward a proinflammatory state that favors tumor progression.

Other gut microbiota metabolites, such as tryptophan, activate the AHR, a transcription factor regulating gene expression, in GAMs. AHR activation affects astrocytes by regulating nerve excitability and decreasing T cell-dependent inflammation in the CNS.^10^ In GB, tumor-derived kynurenine activates AHR, driving CD8 T-cell dysfunction and adenosine production, which consequently upregulates the ectonucleotidases CD39 and CD73 in tumor-associated macrophages.^22^ Elevated kynurenine levels correlate with increased Treg recruitment and cytotoxic T-cell dysfunction, allowing GB cells to evade immune surveillance.^27^ Additionally, microbial lipopolysaccharides (LPS), derived from Gram-negative bacteria, trigger chronic inflammation through toll-like receptor 4 (TLR4) activation, leading to NF-κB-driven cytokine storms that enhance glioma proliferation and angiogenesis.^31^ The presence of dysbiosis-induced LPS has also been linked to increased infiltration of immunosuppressive macrophages, further fueling tumor growth and therapy resistance.

In contrast, secondary bile acids (eg lithocholic acid and deoxycholic acid) and polyamines (eg spermidine, putrescine) have been implicated in reducing inflammation and modulating tumor metabolism. Bile acids activate the farnesoid X receptor, inhibiting NF-κB and STAT3 activation, thereby suppressing glioma-associated inflammation.^74^ Polyamines, while possessing antiinflammatory properties through autophagy activation, also support glioma proliferation in certain contexts by promoting angiogenesis and metabolic adaptation.^75^ Thus, targeting these metabolites through dietary interventions, probiotic supplementation, or microbiome-modulating therapies may provide novel immunomodulatory strategies for GB treatment.

Collectively, the gut microbiome plays an active role in shaping the inflammatory landscape of GB, either suppressing or promoting tumor progression depending on microbial composition and metabolite availability. The dysregulated production of SCFAs, kynurenine, LPS, bile acids, and polyamines due to gut microbiota imbalance not only alters immune cell function but also influences glioma growth, immune evasion, and therapy resistance. Understanding these complex interactions may open new therapeutic avenues, including microbiota-targeted interventions to restore homeostasis and enhance antitumor immunity in GB patients.

### Neurotransmitters

Neurotransmitters are chemical messengers crucial for neuronal communication, and recent evidence suggests that the gut microbiota influences neurotransmitter levels, impacting GB progression.

#### Dopamine

Gut microbiota, through microbial metabolism of tyrosine, affects dopamine,^76^ which regulates mood, motivation, and reward. Dysregulation of dopaminergic signaling is noted in GB, where overexpression of dopamine receptor 2 (DRD2) enhances glioma cell proliferation and migration.^10^^,^^77^ Microbes like *Lactobacillus* and *Bifidobacterium* modulate dopamine levels, and their reduction in dysbiosis may worsen glioma progression. Targeting DRD2 with antagonists is a potential therapeutic strategy.^77^

#### Serotonin

Nearly 90% of serotonin is synthesized in the gut, with gut microbiota playing a regulatory role.^76^^,^^78^ Elevated serotonin levels have been linked to enhanced glioma cell proliferation and angiogenesis, supplying essential nutrients to tumors. Species such as *Enterococcus* and *Streptococcus* influence serotonin biosynthesis,^79^ and microbial imbalances could promote glioma growth.

#### Norepinephrine

Involved in stress responses and blood flow regulation, norepinephrine also has immunomodulatory effects in GB. Gut microbiota influences its biosynthesis^80^ and dysbiosis can lead to norepinephrine reduction, contributing to immunosuppression in the tumor microenvironment. Restoring norepinephrine or enhancing its signaling could improve antitumor immune responses.^59^

#### Gamma-Aminobutyric Acid

GABA is the primary inhibitory neurotransmitter in the CNS. Gut bacteria like Bifidobacterium and Lactobacillus produce GABA,^76^^,^^81^ and reductions in these microbes have been linked to increased glioma progression. Disruption of GABA signaling affects glioma cell migration and multiplication, with GABA receptors as potential therapeutic targets.^82^

#### Glutamate

This primary excitatory neurotransmitter is involved in cognitive processes and synaptic plasticity. Excessive glutamate release in GBM leads to excitotoxicity, damaging neurons and promoting tumor migration. Gut microbiota influences glutamate metabolism,^76^ and dysbiosis may exacerbate neurotoxicity and tumor spread. Inhibiting glutamate release or targeting its receptors could constrain tumor proliferation.^77^

#### Acetylcholine

A neurotransmitter involved in muscle activation, memory, and learning, altered acetylcholine signaling in GB affects tumor cell proliferation and migration.^59^ Certain bacteria such as *Bacillus spp.* produce acetylcholine precursors,^83^ and disruptions in cholinergic signaling may contribute to tumor progression.

## The Role of Antibiotics in GB Development

The role of antibiotics in GB development and progression is still unclear, with studies presenting contradictory findings. Some studies suggest that antibiotics may exacerbate GB progression by disrupting the gut microbiota and promoting an immunosuppressive environment. For example, D’Alessandro et al.^7^ found that GL261 mice treated with antibiotics showed increased tumor growth, altered gut microbiota composition, including increase in *Burkholderiales* and decrease in *Prevotellaceae*, reduced cytotoxic NK cells and altered expression of inflammatory proteins in microglia. These changes may explain the increased glioma growth observed.^57^ These findings imply that chronic antibiotic use could potentially predispose individuals to glioma growth by disrupting the gut–brain axis and immune responses.

In contrast, other studies report no significant association between antibiotic use and glioma risk. For example, in a case control study by Haedenkamp et al.^4^ no evidence was found that antibiotics increase the risk of developing glioma. Additionally, some antibiotics have been associated with antitumor effects. Hu et al.showed that the antibiotic Clofoctol reduced glioma growth *in vivo* and *in vitro* by upregulating the tumor suppressor gene *KLF13* (Krüppel-like Factor 13).^85^ Similarly, salinomycin has shown antitumor effects in depleting GB stem cells *in vitro*, though its neurotoxicity requires further research to ensure safety.^86^

Antibiotics may influence GB development through various mechanisms. They can disrupt gut microbiota composition and diversity, leading to dysbiosis, which in turn affects immune responses and may promote an immunosuppressive environment favoring tumor growth. Dysbiosis may also cause pathogenic bacteria overgrowth, triggering proinflammatory cytokines and metabolites that contribute to tumor progression. Additionally, gut microbiota changes can impair immune cell differentiation and function, such as reducing cytotoxic NK cells,^57^ weakening antitumor immune responses, and enabling GB cell proliferation. Imbalances in proinflammatory and antiinflammatory cytokines, such as elevated IL-6 and TNF-α cytokines, may further promote tumor proliferation and angiogenesis. Lastly, antibiotics can also affect the integrity of the BBB directly or indirectly via microbiota changes, facilitating immune cell and metabolite infiltration into the CNS, potentially influencing the tumor microenvironment and promoting GB progression.^57^^,^^87^

While more studies are needed to conclusively determine the effect of antibiotics on GB progression, caution is advised when considering antibiotics for GB treatment. Antibiotics have been shown to decrease cognition, increase mortality rates and cause severe colitis in mice.^88^ They are also nonspecific and can reduce overall bacterial diversity.^18^ Further research is needed to clarify the mechanisms by which antibiotics affect GB progression, and to determine the clinical implications of antibiotic use in GB patients.

## The Role of the Microbiota in GB Treatment

GB is typically managed with a combination of surgery, radiotherapy and chemotherapy. However, these treatments are often ineffective, and GB remains difficult to treat.^10^ Microbiota-based therapies offer promising avenues for enhancing the efficacy of existing GB treatments and can be easily integrated with current treatment modalities.

Emerging evidence suggests that targeting gut microbiota imbalances, or dysbiosis, may offer potential therapeutic benefits in modulating GB progression and enhancing the efficacy of standard and adjuvant therapies. As previously mentioned, the interaction between the gut microbiota and the GB tumor microenvironment involves complex regulatory pathways, including immune modulation, metabolic regulation, neuroendocrine communication, and dietary interventions. Dietary strategies that optimize gut microbiota, such as those rich in dietary fiber and antioxidants, can enhance responses to chemotherapy, radiation, or ICIs by reducing treatment toxicity and improving efficacy. The gut microbiota’s involvement in drug metabolism further highlights its influence on the bioavailability and efficacy of antitumor drugs. Therapeutic approaches such as fecal microbiota transplantation (FMT) aim to restore microbiota balance and enhance antitumor immunity in GB patients. Additionally, probiotics and prebiotics may promote the growth of beneficial bacteria, offering a supportive role in improving TME and treatment outcomes.

These insights into the gut–brain axis and microbiota interactions are paving the way for novel strategies in GB therapy, supporting the integration of microbiota modulation with existing treatment methods to potentially improve patient survival and therapeutic response.

### Temozolomide and Microbiota

TMZ, a common oral chemotherapeutic agent for GB treatment, alters the bacterial composition in mice and humans with glioma, potentially affecting anticarcinogenic microbes. Patrizz et al.investigated how glioma progression and TMZ chemotherapy influence gut microbiota composition in mice and humans.^6^ The findings indicate that glioma itself reduces microbial diversity and alters bacterial populations, while TMZ further exacerbated dysbiosis by depleting beneficial bacteria. Similar gut microbiota changes were observed in fecal samples from glioma patients, highlighting the disruption of microbial homeostasis by both glioma and its treatment. These findings emphasize the bidirectional relationship between gut microbiota and GB progression, suggesting that targeting the gut microbiome could have implications for managing glioma patients.

Hou et al.^9^ investigated the role of gut microbiota in modulating the effectiveness of TMZ therapy for GB patients. The researchers found that variations in gut microbiota composition among individuals can influence the immune system’s response to TMZ, thereby affecting its therapeutic efficacy. Specifically, certain microbial profiles were associated with enhanced immunomodulatory effects, leading to improved treatment outcomes.

These findings suggest that the gut microbiota plays a significant role in determining individual responses to TMZ therapy, highlighting the potential for microbiome-targeted interventions to optimize GB treatment. Given the growing recognition of the gut–brain axis in neuro-oncology, targeting microbiota dysbiosis could offer new therapeutic opportunities to enhance TMZ efficacy and mitigate its adverse effects. However, more research is needed to understand the implications of gut microbiota on GB development and TMZ efficacy.^7^^,^^90^

### Immune Checkpoint Inhibitors

When exposed to a threat, our immune system unleashes various mechanisms to deal swiftly with the invaders while at the same time protecting and conserving self-tolerance in cells; this is mainly through various checkpoints that regulate the immune system. One of these checkpoints is the programmed cell death protein 1 *(PD-1)*, which plays a crucial role in immune system homeostasis. Tumor cells can evade immune surveillance and suppress the immunity by targeting the PD-1 checkpoint protein. As a result, focus is being shifted to checkpoint blockades, where monoclonal antibodies act as anti-PD-1 agents which in theory creates antitumor immune responses.^91^ Moreover, ICIs target immune cell inhibitory receptors but have not yielded positive clinical results as monotherapy for GB.^12^ The CheckMate 143 trial, which was the first major trial for immunotherapy in GB, showed no significant difference between GB patients treated with anti-PD1 monotherapy and those receiving standard care.^92^ Therefore, combination therapies are considered necessary for the management of GB.

Traditionally regarded as an immune-privileged organ, the brain’s capacity to engage in immune responses, particularly in the context of GB, is increasingly recognized. While GB is typically characterized by its immunosuppressive environment, studies have demonstrated that ICI therapies can induce antitumor immune responses involving CD4+ T cells and phagocytic microglia, enhancing treatment efficacy. Recent insights into the role of gut microbiota in modulating immune responses have opened new avenues for enhancing ICI therapy in GB. Specific gut microbiota were shown to bolster CD8+ T cell function, enhancing antigen presentation and immune response within the tumor microenvironment.^93^ Thus, the gut microbiota emerges as a critical player in this context, influencing the effectiveness of ICIs through the gut–brain axis, as ICI efficiency may be enhanced by manipulating the gut microbiota to reduce dysbiosis.

Oral supplementation of *A. mucinophilia* following fecal microbiota transplantation (FMT) restored PD-1 blockade sensitivity in epithelial cancer patients resistant to ICI treatment. While not specific to GB, this highlights the therapeutic potential of microbiome modulation for treatment-resistant gliomas.^94^ Dees et al.demonstrated *in vivo* how microbiome changes influence GB management.^58^ Gnotobiotic mice colonized with human microbial communities from healthy donors showed varied responses to anti-PD1 therapy. *Bacteroides cellulosilyticus* was abundant in the 2 mouse lines that responded to anti-PD1 with increased survival, suggesting microbiome interactions influence immunotherapy efficacy in GB.^58^ Key gut-derived metabolites like inosine and SCFAs further illustrate these effects. Inosine enhances tumor antigen presentation and boosts T cell-mediated cytotoxicity,^95^ while SCFAs, such as propionic acid, regulate immune cell metabolism and improve ICI efficacy.^96^ A recent study using an orthotopic mouse GB model revealed SCFA supplementation counteracted antibiotic-induced dysbiosis, which had reduced SCFA levels and increased tumor-associated macrophages.^75^ SCFA supplementation promoted a shift toward antiumor macrophages via glycolysis activation, enhancing immune responses against GB marked by increased proinflammatory cytokines such as IFN-γ, IL-2, and TNF-α, and reduced immunosuppressive cytokines like IL-10. These findings suggest that targeting gut dysbiosis and restoring SCFA levels could improve GB treatment outcomes by enhancing antitumor immune responses.

These findings underscore the potential of leveraging gut microbiota to overcome GB’s immunosuppressive barriers and improve ICI therapy effectiveness, offering promising strategies for future therapeutic interventions.

FMT, which involves the transfer of fecal matter from a healthy donor to a patient, is one such method of manipulating gut microbiota to restore balance. FMT has shown potential in enhancing the efficacy of ICIs by modulating the gut microbiota to favor beneficial bacteria. For instance, the presence of *Akkermansia muciniphila* has been associated with better responses to ICIs.^94^ Integrating FMT with ICI therapy could improve the immune response in GB patients, making tumors more susceptible to immunotherapy.^94^

### Genetically Engineered Bacteria

Genetically modified anaerobic bacteria such as *Salmonella*, *Escherichia coli*, and *Streptococcus pyogenes* can be engineered to deliver therapeutic agents directly to the GB tumor microenvironment. These bacteria can target hypoxic and necrotic regions of GB inaccessible to conventional drugs and by delivering toxins, antibodies, and cytokines in these regions, they can enhance the antitumor immune response and inhibit tumor growth.^10^^,^^97^

### Probiotics

Probiotics are live microorganisms associated with numerous health benefits, including enhanced human immune response.^98^ They have gained popularity for their potential effects on cancer prevention and development. For example, probiotics have been used as adjuvant therapy in colorectal cancer^99^ and shown to regulate neuroendocrine homeostasis.^18^ In GB, probiotics have demonstrated cytotoxic activity against GB cells. An *in vitro* study by Fatahi and colleagues found that Kefir, a milk fermented with *Lactobacillus* and *Bifidobacterium,* had cytotoxic effects on GB cell lines.^100^ While promising, more trials are needed to investigate the potential of probiotics in GB management.

Bacterial components, including toxins, have also shown promise in GB management. Cytotoxic necrotizing factor 1 (CNF1) from *E. coli,* decreased tumor volume and improved survival in mice.^97^ Initially, CNF1 delivery was thought to be invasive due to its inability to cross the BBB. However, Vannini et al.created a chimeric protein by conjugating CNF1 with chlorotoxin (CTX-CNF1),^101^ allowing it to cross the BBB and exhibit similar effects as CNF1 in gliomas.^97^^,^^101^ These findings highlight the potential use of bacteria for GB treatment.

### Microbial Metabolites

Microbial metabolites, such as SCFAs and tryptophan metabolites, can modulate immune responses and influence tumor progression. Supplementing these metabolites or promoting their production through dietary interventions could enhance the efficacy of existing GB treatments. For example, SCFAs have antiinflammatory properties and can regulate the differentiation of immune cells, potentially improving the response to chemotherapy and radiotherapy.^35^

## Conclusion

GB is recognized as the most aggressive cancer in the CNS, largely due to its capacity to create an immunosuppressive environment that renders it highly resistant to current therapeutic agents. Understanding the pathogenesis of GB is essential for developing more effective treatments. This review emphasizes the importance of unraveling the mechanisms of GB-induced immunosuppression and highlights how a deeper understanding of the interactions between GB, the immune system, and the microbiota may lead to the discovery of novel therapeutic targets.

The gut–brain axis significantly influences immune responses, brain function and pathology, with emerging evidence linking it to brain tumor development. Intestinal microbiota has been shown to affect the immune system through production of various metabolites, neurotransmitters and cytokines, which either contribute to the development of GB or offer a therapeutic potential, presenting both significant challenges and promising opportunities for therapeutic advancement. However, the mechanisms by which gut dysbiosis influences GB remain unclear. While studies suggest microbial metabolites and immune modulation play roles in the tumor microenvironment, their precise functions are largely unexplored. Additionally, developing microbiota-derived biomarkers for diagnosis, prognosis, or treatment response is still in early stages, requiring robust clinical validation. Interpatient variability in gut microbiome composition further complicates the development of standardized therapeutic interventions, emphasizing the need for personalized approaches in microbiota-based therapies.

Despite these challenges, emerging research highlights several promising therapeutic strategies. FMT and the use of probiotics and prebiotics represent potential methods to beneficially alter microbiota composition, thereby modulating immune responses and enhancing tumor treatment outcomes. The development of microbiota-targeting drugs that influence immune pathways and genetically engineered bacteria capable of delivering therapeutic agents to tumors could overcome barriers like the BBB and improve treatment precision. Modulating the gut microbiome to enhance ICI efficacy also shows significant potential, given the microbiome’s role in systemic immune regulation. Identifying bacterial strains that influence GB progression may lead to probiotics that improve chemotherapy and radiotherapy by altering the tumor microenvironment and reducing immunosuppression.

The discovery and characterization of specific bacterial strains that influence GB disease progression could lead to probiotic supplements specifically designed for GB patients, improving the effectiveness of chemotherapy and radiotherapy by altering the tumor microenvironment, reducing immunosuppression and making cancer cells more vulnerable. Furthermore, nutritional therapy may play an important role in the treatment of GB given the influence of food on the intestinal microbiota. Research focusing on finding dietary patterns in support of microbiome that is beneficial for GB treatment could result in tailored dietary suggestions for GB patients.

Collectively, these strategies highlight the transformative potential of microbiota-based interventions in managing GB, though continued research is essential to translate these discoveries into clinical practice. Understanding the interactions between the gut microbiota, immune system, and GB can lead to the development of novel therapeutic strategies that improve patient outcomes. Future research should focus on elucidating the mechanisms underlying these interactions, optimizing microbiota-based therapies, and conducting rigorous clinical trials to validate their efficacy. By integrating microbiota-based therapies with existing treatments, the effectiveness of GB management can be enhanced and the prognosis for patients with this aggressive disease can be improved.
